# Metallothionein-I/II Knockout Mice Aggravate Mitochondrial Superoxide Production and Peroxiredoxin 3 Expression in Thyroid after Excessive Iodide Exposure

**DOI:** 10.1155/2015/267027

**Published:** 2015-05-25

**Authors:** Na Zhang, Lingyan Wang, Qi Duan, Laixiang Lin, Mohamed Ahmed, Tingting Wang, Xiaomei Yao

**Affiliations:** ^1^Department of Pathophysiology, School of Basic Medical Sciences, Tianjin Medical University, Tianjin 300070, China; ^2^Key Lab of Hormones and Development of Ministry of Health, Institute of Endocrinology, Metabolic Disease Hospital, Tianjin Medical University, Tianjin 300070, China

## Abstract

*Purpose*. We aim to figure out the effect of metallothioneins on iodide excess induced oxidative stress in the thyroid. *Methods*. Eight-week-old MT-I/II knockout (MT-I/II KO) mice and background-matched wild-type (WT) mice were used. Mitochondrial superoxide production and peroxiredoxin (Prx) 3 expression were measured. *Results*. In in vitro study, more significant increases in mitochondrial superoxide production and Prx 3 expression were detected in the MT-I/II KO groups. In in vivo study, significantly higher concentrations of urinary iodine level were detected in MT-I/II KO mice in 100 HI group. Compared to the NI group, there was no significant difference existing in serum thyroid hormones level in either groups (*P* > 0.05), while the mitochondrial superoxide production was significantly increased in 100 HI groups with significantly increased LDH activity and decreased relative cell viability. Compared to WT mice, more significant changes were detected in MT-I/II KO mice in 100 HI groups. No significant differences were detected between the NI group and 10 HI group in both the MT-I/II KO and WT mice groups (*P* > 0.05). *Conclusions*. Iodide excess in a thyroid without MT I/II protection may result in strong mitochondrial oxidative stress, which further leads to the damage of thyrocytes.

## 1. Introduction

Iodine is a fundamental constituent of thyroid hormones and has stimulatory effects on hydrogen peroxide (H_2_O_2_) generation, which is involved in oxidative stress [1, 2]. 150 *μ*g iodine is the daily requirement for thyroid hormone synthesis [3]. The International Council for the Control of Iodine Deficiency Disorders proposed that 150–299 *μ*g/day is adequate to cover the thyroid requirement, and the maximal allowable dietary dose of iodine is 1.0 mg/day for children and 2.0 mg/day for adults [4]. However, concentrations of iodine can be up to several hundredfold to thousandfold higher than the above amounts [5]. Intakes of up to 600 mg per day in the European Union and 1100 mg per day in the United States are declared as tolerable for adults [6]. Iodide excess has been demonstrated to be toxic to the thyroid [7–10] and may lead to hypothyroidism, hyperthyroidism, euthyroid goiter, or thyroid autoimmunity [3, 11–13], with oxidative stress being one of the underlying mechanisms [14, 15]. Animal models with different iodine intake dietary were established in mice [16–18] and in rats [19, 20] to study the effects of excess iodine on the thyroid. Humans may get exposed to excess iodine through medications or diet. Substances involved in iodine excess include iodine-containing drugs, amiodarone being the prime offender. Approximately 9 mg of iodine is released following a daily dose of 300 mg amiodarone. Other substances include iodpovidone used for topical disinfection and seaweed. Seaweed preparations containing up to 2 mg iodine per gram in protein-bound form and may cause dietary iodine intake to exceed 5000 mg per day in populations with increased consumption [21, 22]. Contrast agents for conventional radiography or computer tomography may also lead to iodine excess. Radiologists use up to 100 g or more of iodine in complex organic molecules. Erythrosine, a food additive used to improve the colour of canned cherries or of candies, contains 57% iodine [2, 3]. Although iodized salt programs have made great progress in the global effort to control iodine deficiency, they need to be carefully monitored to ensure adequate iodine intake while avoiding iodine excess [20].

The mitochondria contain specific receptors for thyroid hormones and are an important source of reactive oxygen species (ROS) [22]. Thyrocytes possess various enzymatic systems such as peroxidise (GPx), catalases, superoxide dismutases (SOD), and peroxiredoxins (Prxs) that contribute to limiting cellular injuries [23]. Peroxiredoxin 3 (Prx 3) is predominantly located in the mitochondria and is responsible for scavenging ROS. Prx 3 can be the target for up to 90% of hydrogen peroxide generated in the matrix [24]. Metallothioneins (MTs) have been demonstrated to have strong antioxidative activity in protecting cells from the damaging effects of ROS. They too are regarded as scavengers of ROS, including superoxide and hydroxyl radicals [25, 26].

In light of previous works documented on iodide induced oxidative stress and thyrotoxicity, we have demonstrated the time-course and concentration-response effects of early acute high concentrations of iodide exposure on mitochondrial superoxide generation. When endogenous antioxidant systems were overwhelmed by high concentrations of iodide induced ROS production, they began attacking proteins, lipid, and nucleic acids [15]. However the nature of iodide excess induced oxidative stress in thyroid with or without MT I/II remains to be determined.

By targeting mitochondrial superoxide production and Prx 3 protein expression, we aimed to figure out the effect of high concentrations of iodide on mitochondrial superoxide production and Prx 3 expression in the thyroids of metallothionein I/II knockout (MT-I/II KO) mice in vitro and in vivo. We propose that iodide excess in a thyroid without MT I/II protection may result in strong mitochondrial oxidative stress, which further leads to the damage of thyrocytes.

## 2. Methods

### 2.1. Animals and Thyroid Cell Suspension Preparation

Eight-week-old 129S7/SvEvBrd-Mt1^tm1Bri^Mt2^tm1Bri^/J (MT-I/II knockout, MT-I/II KO) mice (number 002211, Jackson Lab, Maine, USA) and background-matched wild-type (WT) mice were used. In the in vitro study, a thyroid cell suspension was prepared. A cell suspension (5 × 10^4^ cells/well) was incubated in medium RPMI-1640 with 10% fetal bovine serum (FBS) at 37°C in an atmosphere comprising 5% CO_2_ and 95% air and exposed to high concentrations of potassium iodide (KI) or 10^−3^ M H_2_O_2_ for 2 hours. In vivo, MT-I/II KO and WT mice were randomly divided into control groups (normal iodide intake, NI), 10 times iodide intake groups (10 HI), and 100 times iodide intake groups (100 HI). NI groups received deionized water and a normal diet (daily iodide uptake is 1.5 *μ*g/d). In the 10 HI and 100 HI groups, 15 *μ*g/d and 150 *μ*g/d KI were added, respectively, to the deionized water for 14 days before sacrifice. Animal procedures were approved by the Institutional Animal Care and Use Committee of Tianjin Medical University and in accordance with the NIH Guide.

### 2.2. Reagents

MitoSOX Red (3,8-phenanthridinediamine, 5-(6′-triphenylphosphoniumhexyl)-5,6-dihydro-6-phenyl) was purchased from Invitrogen (Invitrogen Life Technologies, CA, USA). Anti-Peroxiredoxin 3 antibody was purchased from Abcam (Abcam, Cambridge, MA, USA). *β*-Actin Rabbit mAb was purchased from Cell Signaling (Cell Signaling Technology, Inc., MA, USA). 3-(4,5-Dimethylthiazol-2-yl)-2,5-diphenyl-tetrazolium bromide (MTT) and TSH were purchased from Sigma (Sigma-Aldrich, MO, USA). RPMI-1640 and FBS were purchased from GE Healthcare Life Sciences (Hyclone, UT, USA). All the other chemicals made in China were of analytical grade.

### 2.3. Cell Viability Assay

Cell viability was evaluated with the MTT assay. A cell suspension (5 × 10^4^ cells/well) was incubated in medium RPMI-1640 with 10% fetal bovine serum (FBS) at 37°C in an atmosphere comprising 5% CO_2_ and 95% air and exposed to high concentrations of potassium iodide (KI) or 10^−3^ M H_2_O_2_ for 2 hours in order to get a final concentration of 10^−4^ M, 10^−3^ M, and 10^−2^ M. The group without KI is the control group; the well with only medium in is regarded as blank group. Subsequently, 10 *μ*L of 3-(4,5-dimethylthiazol-2-yl)-2,5-diphenyltetrazolium bromide (MTT, 5 mg/mL) was added in dark and incubated for 4 h and covered with aluminium foil. After incubation for 2 hours, the supernatant was removed, and 100 *μ*L of DMSO was added to each well to dissolve the formazan crystals formed and was then shaken for 10 min. Absorbance was measured by a spectrophotometer at 490 nm (Wallac 1420 VICTOR3, PerkinElmer) [33, 34].

### 2.4. Lactate Dehydrogenase (LDH) Assay

LDH release in the supernatant following different treatments was measured using a cytotoxicity detection kit (LDH) (Nanjing Jiancheng Bioengineering Institute, Jiangsu, China). LDH is an oxidoreductase that catalyzes the interconversion of lactate and pyruvate. LDH is a stable cytosolic enzyme that upon membrane damage is released into cell culture media. The assay is based on the reduction of the 2-p-iodophenyl-3-nitrophenyl tetrazolium chloride (tetrazolium INT) to a red formazan which is specifically detected by colorimetric (450 nm) assay.

### 2.5. Flow Cytometry

A mitochondrial superoxide indicator (MitoSOX Red, Invitrogen) was used to measure mitochondrial superoxide production by flow cytometry. In vivo, the thyroid cell suspension of MT-I/II KO and WT mice was prepared for (normal iodide intake, NI) 10 times iodide intake groups (10 HI) and 100 times iodide intake groups (100 HI). For the in vitro study, a thyroid cell suspension was prepared first. The thyroid cell suspension was exposed to high concentrations of potassium iodide (KI) (10^−4^ M, 10^−3^ M, and 10^−2^ M) or 10^−3^ M H_2_O_2_ for 2 hours. Subsequently, 5 *μ*M of MitoSOX was added and incubated for 10 min at 37°C in the dark. Then the cells were washed with Hank's solution and were suspended in Hank's solution with 1% BSA. The fluorescence intensity of MitoSOX was detected by a FACSCalibur (BD Bioscience, San Jose, CA), and the excitation/emission wavelength is 488 nm/575 nm. Collecting FL2 channel forward scattering (forward scatter, FSC) and lateral scattering (side scatter, SSC) data, 10000 cells were collected for each sample. The control group without MitoSOX was regarded as the blank zero group for standardization [35–38].

### 2.6. Western Blot Analysis

Whole cell proteins were assayed for protein concentration using the bicinchoninic acid (BCA) protein assay kit (Beyotime Institute of Biotechnology, Jiangsu, China). 50 *μ*g of protein was transferred to nitrocellulose membrane followed by SDS polyacrylamide gel electrophoresis. The nitrocellulose membrane was incubated overnight with primary antibodies: Anti-Peroxiredoxin 3 antibody (Abcam, Cambridge, MA, USA) and horseradish peroxidase (HRP) conjugated secondary antibody, developed by Immobilon Western Chemiluminescent HRP Substrate (Merck Millipore, MA, USA). To verify equal loading, *β*-actin (1 : 1000) (Cell Signaling Technology, Inc., MA, USA) was used as a loading control. Blots were scanned as grayscale images and quantitated using Image J software (NIH). All the blot intensities were normalized with that of loading control *β*-actin [39].

### 2.7. Determination of Urinary Iodine Concentrations and Serum Thyroid Hormones

Spot urine samples of mice were collected and urinary iodine concentrations were measured by As-Ce catalytic spectrophotometry in the Key Lab of Hormones and Development Ministry of Health, Institute of Endocrinology, Tianjin Medical University [40]. Blood was obtained 14 days after NI, 10 HI, or 100 HI intake. Samples of blood were centrifuged for 10 min; serum samples were obtained and stored at −80°C for use. Serum concentrations of thyroxine (T_4_), free thyroxine (FT_4_), triiodothyronine (T_3_), and free triiodothyronine (FT_3_) were determined by using the Direct Chemiluminescence Technology Competitive Immunoassay Kits (Siemens Healthcare Diagnostic, Inc.).

### 2.8. Statistics

The data was represented as mean ± SD. Based on the Kolmogorov-Smirnov (K-S) for normality test and Levene statistic for the test of homogeneity of variances. Urinary iodine concentrations were expressed as the median and determined with the nonparametric Kruskal-Wallis test; one-way ANOVA with the least significant difference (LSD) test was performed using SPSS 17.0 to determine the differences between the groups. A *P* value of less than 0.05 was considered to be statistically significant.

## 3. Results

### 3.1. Effect of MT-I/II on High Concentrated Iodide Exposure Induced Mitochondrial Superoxide Production and Prx 3 Protein Expression In Vitro

**Figure 1:**
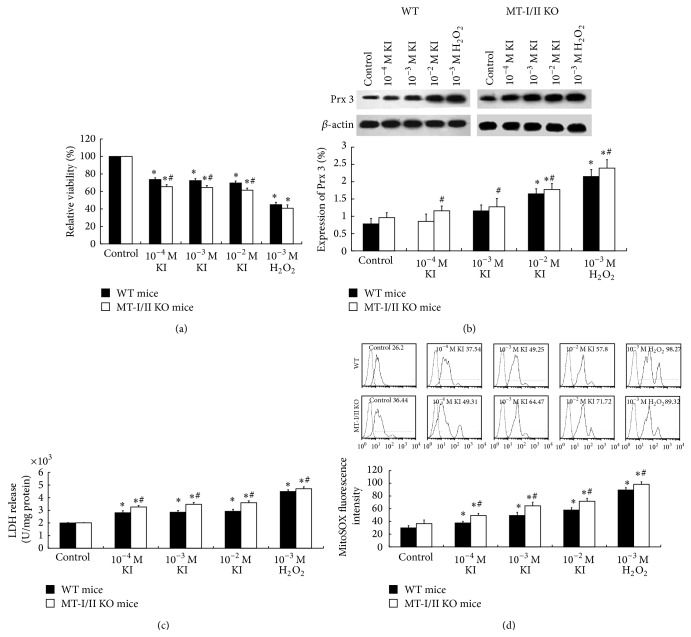
Oxidative and antioxidative effect of high concentrations of KI on the mitochondria of thyrocytes in MT-I/II KO mice and WT mice. (a) Decreased relative viability induced by high concentrations of KI (10^−4^ M, 10^−3^ M, and 10^−2^ M) or 10^−3 ^M H_2_O_2_ in the thyroid cells of MT-I/II KO mice by MTT assay (*N* = 8). (b) Representative western blot of Prx 3 (27 kDa), *β*-actin (45 kDa) was used as a loading control. Densitometric analysis showed a significant increase (^*^ *P* < 0.05) in Prx 3 expression compared to untreated control in the thyroid cells of either WT or MT-I/II KO mice. (c) Increased LDH release following 2 h of high concentration of KI or H_2_O_2_ exposure in the thyroid cells of MT-I/II KO mice (*N* = 8). (d) High concentration of KI or H_2_O_2_ induced increased mitochondrial superoxide production in the thyroid cells of WT and MT-I/II KO mice. Histogram analysis was performed on the mean fluorescence intensity of MitoSOX Red as measured by flow cytometry. Experiments were repeated 3 times with similar results. Data are represented as mean ± SD. One-way ANOVA with the LSD test was used. ^*^ *P* < 0.05 compared with the control group of WT or MT-I/II KO mice, respectively; ^#^ *P* < 0.05 WT mice versus MT-I/II KO mice under the same treatment.

To investigate the antioxidative role of MT-I/II in oxidative stress induced by high concentrations of iodide in the thyroid, thyroid cell suspensions were prepared from the thyroid of MT-I/II KO mice and WT mice. The cells were then exposed to high concentrations of KI (10^−4^ M, 10^−3^ M, and 10^−2^ M) or 10^−3^ M H_2_O_2_ for 2 hours. Following 10^−4^ M, 10^−3^ M, and 10^−2^ M of KI or 10^−3^ M H_2_O_2_ exposure, we showed a significant increase in mitochondrial superoxide production with increased MitoSOX Red fluorescence intensity (*P* < 0.05) (Figure 1(d)) and an increase in LDH release (*P* < 0.05) (Figure 1(c)) with a decrease in relative cell viability (*P* < 0.05) (Figure 1(a)) in the thyroid cell suspensions of both MT-I/II KO and WT mice groups. Compared to WT mice group, a more significant increase of mitochondrial superoxide production, increase in LDH release, and decrease in a relative cell viability can be detected in MT-I/II KO mice group (*P* < 0.05) (Figures 1(d), 1(c), and 1(a)). Compared to control group, the Prx 3 protein expressions were significantly increased in 10^−2^ M KI or 10^−3^ M H_2_O_2_ exposure group in both MT-I/II KO and WT mice groups (*P* < 0.05). Compared to WT mice group, there was a significant increase of Prx 3 protein expression in the thyroid cell suspensions of MT-I/II KO mice with the increase of KI concentration (*P* < 0.05) (Figure 1(b)).

### 3.2. Urinary Iodine Concentrations In Vivo

In order to verify the model of iodine excess in vivo, urinary iodine concentrations were detected after the MT-I/II KO mice and WT mice had NI, 10 HI, and 100 HI for 14 days. The median urinary iodine concentrations in NI, 10 HI, and 100 HI were shown in Table 1. The high iodide intake of 10 HI group and 100 HI group in both MT-I/II KO mice and WT mice resulted in significant increases in urinary iodine concentration when compared to theNI group (*P* < 0.05). The urinary iodine concentrations were increased as the iodine supply increased to 10 HI, 100 HI in both WT and MT-I/II KO mice. Moreover, compared to 10 HI group, the extent of increase in urinary iodine concentration in 100 HI was significantly higher (*P* < 0.05). Compared to WT mice, MT -I/II KO mice had a more prominent increase of urinary iodine concentration in 100 HI group (*P* < 0.05).

### 3.3. Changes of Serum Thyroid Hormones Level in WT and MT-I/II KO Mice following NI, 10 HI, or 100 HI Intake for 14 Days

We demonstrated that, compared to the NI group, there was no significant difference existing in serum thyroid hormones level (T_3_, T_4_, FT_3_, and FT_4_) in either 10 HI group or 100 HI group in both WT mice and MT-I/II KO mice (*P* > 0.05). However, compared to WT mice, there was a significant difference in serum thyroid hormones level (FT_4_) in 100 HI group in MT-I/II KO mice (*P* < 0.05) (Table 2).

### 3.4. High Iodide Intake Induced Increased Mitochondrial Superoxide Production and Peroxiredoxin 3 Expression in Thyroid In Vivo

**Figure 2:**
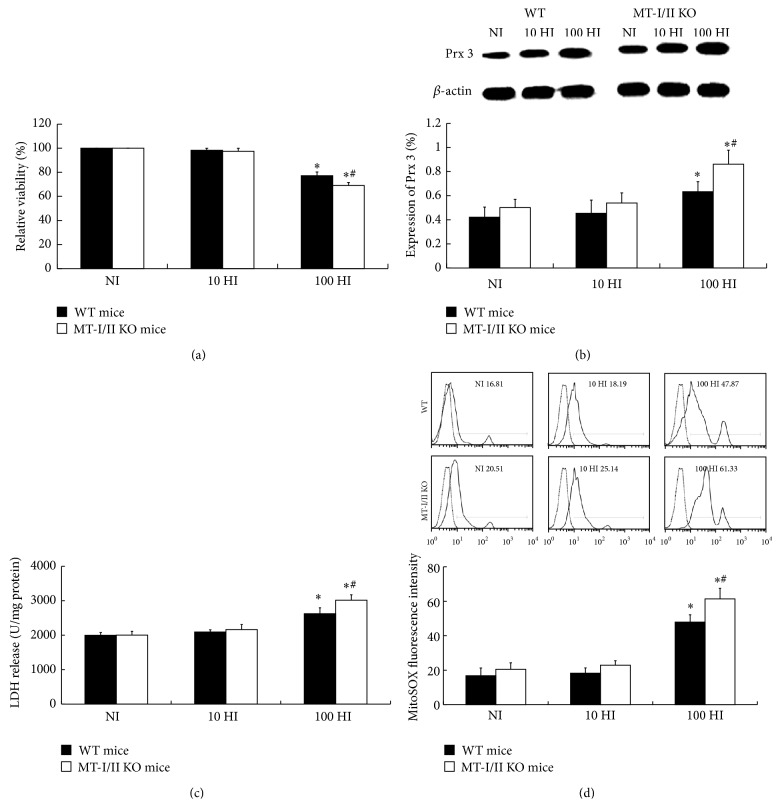
Mitochondrial superoxide production, Prx 3 protein expression, and LDH release and relative viability in the thyroid of WT and MT-I/II knockout mice following NI, 10 HI, or 100 HI diet for 14 days. (a) 100 HI intake decreased the relative viability in thyroid of MT-I/II KO and WT mice. (b) Representative western blot of Prx 3 (27 kDa), *β*-actin (45 kDa) was used as a loading control. Densitometric analysis showed a significant increase in Prx 3 expression in 100 HI group in the thyroid of either WT or MT-I/II KO mice, especially in MT-I/II KO mice. (c) 100 HI increased the LDH release in the thyroid of MT-I/II KO and WT mice. (d) 100 HI intake increased the mitochondrial superoxide production in thyroid of WT and MT-I/II KO mice. Histogram analysis was performed on the mean fluorescence intensity of MitoSOX Red as measured by flow cytometry. Experiments were repeated 3 times with similar results. Data are represented as mean ± SD (*N* = 10/group). One-way ANOVA with the LSD test was used. ^*^ *P* < 0.05 compared with the control group of WT or MT-I/II KO mice, respectively; ^#^ *P* < 0.05 WT mice compared with the MT-I/II KO mice under the same treatment.

To clarify the effect of iodide excess on mitochondrial superoxide production and Prx 3 protein expression in thyroid of MT-I/II KO mice in vivo, mice were divided into normal iodide intake (NI), 10 times iodide intake (10 HI), and 100 times iodide intake (100 HI) groups. Compared to the NI group, the mitochondrial superoxide production measured by flow cytometry, Prx 3 protein expression detected by western blot analysis, and LDH release in 100 HI group were significantly increased in both MT-I/II KO and WT groups (*P* < 0.05). Compared to WT group, more significant increases in the mitochondrial superoxide production, Prx 3 protein expression, and LDH release were detected in MT-I/II KO group (*P* < 0.05). There were no significant differences between the NI group and 10 HI group in both MT-I/II KO and WT mice (*P* > 0.05). Compared with the NI group, relative cell viability of 100 HI group decreased in both the MT-I/II KO and WT mice groups (*P* < 0.05). Compared to the WT group, a more significant decrease in relative cell viability was detected in MT-I/II KO group (*P* < 0.05). No significant difference of relative cell viability was detected between NI group and 10 HI group in both MT-I/II KO and WT mice groups (*P* > 0.05) (Figure 2).

## 4. Discussion

Iodide excess has been recognized as a risk factor for the development of thyroid disease in humans and animals [7–13]. One of the underlying mechanisms is that the increased oxidative stress induced by iodide excess cannot be balanced by endogenous antioxidant systems [14, 15]. In the present study, we focused the research on excessive iodide induced mitochondrial superoxide production and Prx 3 expression in the thyroid with or without MT I/II in vitro and in vivo.

In a previous study [15], we showed that the concentration response of KI induced a decrease in cell viability in FRTL cells by MTT assay; compared to the control group, the relative viability of 10^−5^ M, 10^−4^ M, 10^−3^ M, and 10^−2^ M KI exposure groups was significantly decreased at 24 h. However, no significant decrease in the 10^−6^ M or 10^−7^ M KI exposure groups was observed. A strong stimulatory effect of iodide was detected in 10^−4^ M KI and in 10^−3^ M KI exposure groups at the 2 h time point [15]. Iodide had a stimulatory effect on H_2_O_2_ generation and, when increased, H_2_O_2_ is considered as a limiting step in the biosynthesis of thyroid hormones [1]. The normal relative cell viability in the 10^−6^ M or 10^−7^ M KI exposure groups can be explained by the physiological effect of iodide, which is necessary for the biosynthesis of thyroid hormones [15]. We used KI concentrations of 10^−4^ M and above in the in vitro study and NI, 10 HI, and 100 HI in the in vivo study. In bovine thyroid slices, the maximal stimulation effect on H_2_O_2_ generation was obtained after 2 h of preincubation with 10^−4^ M KI [1]. It was reported that, in vitro, the acute toxic effects of high iodide doses in human thyroid follicles significantly increased the percentage of necrotic cells in 10^−5^ M and doubled with 10^−3^ M as compared to values measured at 10^−7^ M [10]. Iodide concentrations of 10^−4^ M or greater may inhibit the iodothyronine synthesis and thyroid hormone secretion [41]. The concentrations from 10^−5^ to 10^−3^ M were from 100 to 10,000 times higher than the normal iodine plasma levels estimated to be 10^−7^ M, in euthyroid human beings [42].

In the present study, we detected that, in 100 HI intake group, there is a significant change of FT_4_ in MT-I/II KO mice compared to WT mice; however, the values remain within the normal range in thyroid function. The NI and 10 HI groups in both WT and MT-I/II KO mice were under normal conditions and the thyroid function was not affected. Iodine is essential for the synthesis of the thyroid hormones. In most individuals, the decreased production of thyroid hormones is only transient and resumes after adaptation to the acute Wolff-Chaikoff effect [43]. A detailed analysis reveals a persistent drop of serum T_4_ and T_3_ of 25% and 15%, respectively, and a rise of TSH of 2 mU/L. Although no clinical signs of thyroid dysfunction or goiter were found, sonographic thyroid volume is slightly increased [2, 29–32, 44, 45]. Exposure to high concentrations of iodine may decrease the release of thyroid hormone shown by the increase in the serum level of TSH [27, 28, 46] (Table 3).

Thyroid toxicity of iodide excess has been demonstrated in vitro and in animals fed with an iodide-rich diet [42]. We showed that high concentrated iodide induced cytotoxicity in thyrocytes, significantly increasing mitochondrial superoxide anion production in the thyroid in high concentrated iodide exposure in vitro and in 100 HI diet in vivo, in both MT-I/II KO and WT mice, which leads to the decreased relative cell viability in the thyroid. It is suggested that oxidative stress is the underlying mechanism in iodide excess induced apoptosis in thyroid cells [42]. Consistent with previous reports [42, 47–49], we showed that there is a strong increase of superoxide anion production in FRTL cells following high concentrations of iodide exposure [15]. Serrano-Nascimento et al. also observed that iodide excess increased ROS production in thyrocytes and mitochondria were the source of superoxide anion production [50]. The oxidative attack can be explained by the oxidative effect of HI diet on the thyroid gland. ROS include free radicals, such as the superoxide anion, hydroxyl radicals, and the nonradical hydrogen peroxide. Whether cells die from oxidative stress induced apoptosis depends on the balance between the generation of oxidant species and the antioxidant system.

In addition, we demonstrated that no significant difference was detected between NI group and 10 HI group in both MT-I/II KO and WT mice groups. This can be explained by the fact that, under basal conditions, thyroid epithelial cells produce moderate amounts of ROS that are physiologically required for thyroid hormone synthesis [14]. Physiologically, mitochondrial ROS generation is primarily due to electrons leaking from the electron transport chain (ETC) to generate superoxide radical. Superoxide from the electron transport chain and other sources is converted by MnSOD to H_2_O_2_, which is then metabolized by Prx 3 to water [47].

Besides, we demonstrated that high concentrated iodide induces significant increased expression of Prx 3, which suggests that the increase of Prx 3 expression following high concentrated iodide exposure or HI diet in the thyroid may be the protective response against high ROS generation. Prx 3 is an indispensable ROS scavenger that protects against oxidative damage and subsequent apoptosis [51–53]. It is reported that Prx 3 has an essential role in regulating oxidation-induced apoptosis and can be a potential therapeutic target in castrate-independent prostate cancer [52]. Iodide had a stimulatory effect on H_2_O_2_ generation in thyroid slices [1]. At high concentrations (above 0.1 mM), H_2_O_2_ induces apoptosis in thyroid cells [1]. When iodide is in excess as compared to tyrosine residues, it reacts with the iodonium cation formed by iodide oxidation to give molecular iodine. Excess molecular iodine then induced apoptosis through generation of free radicals [54]. H_2_O_2_ generation is a limiting step in thyroid hormone biosynthesis; it is essential for iodide oxidation by thyroperoxidase [14, 55, 56]. H_2_O_2_ can be eliminated by Prx 3. In mammals, Prx 3 is targeted to the mitochondrial matrix. It is estimated that Prx 3 will be the target for up to 90% of hydrogen peroxide generated in the mitochondrial matrix [57]; mitochondrial peroxiredoxins (3 and/or 5) can reduce H_2_O_2_ to water through reducing equivalents provided by thiol-containing proteins [58, 59], but the mechanisms involved are unclear [24, 59]. Drechsel and Patel demonstrated that thioredoxin (Trxs)/peroxiredoxin (Prxs) (Prx 3 and Prx 5) is the major contributing system to H_2_O_2_ removal in brain mitochondria [60]. Prxs and Trxs are important liver antioxidant proteins and play important roles in maintaining liver redox homeostasis [61]. It is reported that chronic ethanol exposure increases NADPH oxidase activity and increases the production of superoxide and hydrogen peroxide [62–64]. Thioredoxin scavenges and reduces ROS via peroxiredoxins [65]. Endoplasmic reticulum stress disrupts the electron transport chain, leading to the increased production of ROS [48, 49]. Bae et al. reported that the antioxidant role of Prx 3 was evident from the findings that pyrazole-induced protein carbonylation and the formation of 4-HNE adducts and MDA in the liver were markedly increased in Prx 3^−/−^ mice compared with wild-type mice [66].

A high urinary excretion of iodine in a spot urine sample is the ultimate proof of iodine excess in vivo [2]. We showed the urinary iodine concentration increased as the iodide supply increased; significantly higher urinary iodine concentration can be detected in 100 HI than 10 HI and in MT-I/II KO mice than in WT mice. This data indicates the adaptation of the thyroid gland to iodine excess and that the excessive iodine is excreted from the urine in order to maintain the normal function of thyroid. This makes the studies in vivo different from studies in vitro. In addition to the urinary excretion of iodine, there are many control mechanisms of the thyroid to counteract iodide excess. These include the sodium-iodide symporter, which controls the limiting step of thyroid hormone synthesis, the Wolff-Chaikoff effect, an arrest of thyroid hormone secretion from stores in the colloid, a preferential secretion of the less active T_4_ over T_3_, and dumping of excess iodine by secreting it in nonhormonal form [2].

Moreover, we demonstrated that significant increases of mitochondrial superoxide production and Prx 3 protein expression can be detected in MT-I/II KO mice group. This data indicated the protective role of MT-I/II against high concentrations of iodide induced mitochondrial superoxide production in the thyrocytes. MT has been demonstrated to play a key role in the detoxification of heavy metals, protecting against various forms of oxidative injury by scavenging oxygen free radicals. MTs belong to the group of intracellular cysteine-rich, metal-binding proteins which is widely expressed in cells (nuclei and/or cytoplasm) of various organs and tumors [67–69]. MTs play a protective role in preventing against intoxication with heavy metals, such as Pb, Hg, Cu, and Cd [67, 70]. All 20 cysteine sulfur atoms are involved in the radical quenching process, and the rate constant for the reaction of hydroxyl radical with MT is about 340-fold higher than that with GSH [71, 72]. There are four MT isoforms in mammals. MT-I and MT-II are mainly involved in the protection of tissue against metal toxicities, oxidative stresses, and apoptosis. It has been demonstrated that the rabbit liver metallothionein-1, which contains zinc and/or cadmium ions, appeared to scavenge free hydroxyl and superoxide radicals [25, 26]. Under low levels of iodide organification in goitrous-modified tissue of thyroid gland, metallothionein may provide a partial compensatory effect on prooxidative processes [73, 74]. Although no cell line or animal model overexpressing MT in the thyroid has been found yet, documented evidence of MT in protecting against oxidative stress in cardiac-specific metallothionein-overexpressed transgenic mice has been demonstrated. Cai et al. reported that metallothionein, as a potent antioxidant, prevented the development of diabetic cardiomyopathy through the inhibition of the mitochondrial cytochrome c-mediated caspase-3 activation pathway [75]. Sun et al. reported that, through doxorubicin induced chronic toxicity in a metallothionein-overexpressed transgenic mouse heart, the antioxidant action of MT is highly responsible for this cardioprotection [76]. Ceylan-Isik et al. reported that the cardiac-specific overexpression of MT rescues LPS-induced cardiac contractile and intracellular Ca^2+^ dysfunctions through the alleviation of ROS/oxidative stress, stress signaling activation, and endoplasmic reticulum (ER) stress [77]. They also reported that the cardiac-specific overexpression of metallothionein protects against nicotine exposure-induced cardiac contractile dysfunction and fibrosis possibly through inhibition of ROS accumulation and apoptosis [78]. Zhang et al. suggest that cardiac overexpression of metallothionein prevents cold exposure-induced cardiac anomalies, possibly through the attenuation of myocardial fibrosis [79]. Hu et al. demonstrated that cardiac-specific overexpression of metallothionein protects against cigarette smoking exposure-induced myocardial contractile and mitochondrial damage, favoring a role of lessened apoptosis in a metallothionein-induced beneficial effect against side-stream smoke exposure [80]. Besides, metallothionein has been shown to regulate apoptosis and proliferation. Overexpression of metallothionein frequently occurs in human tumors and is related to tumor progression [81, 82]. High expression of MT has been observed in different types of cancer and has been considered as a potential prognostic marker in cancers of the breast, pancreas, skin, and cervix [82–86]. Further studies will be attempted to study the oxidative and antioxidative stress on an engineered cell line overexpressing metallothionein.

## 5. Conclusion

The present study provides evidence that high concentrations of iodide may lead to a significant increase in mitochondrial superoxide production and Prx 3 expression in both in vitro and in vivo studies. MT-I/II KO mice aggravated mitochondrial superoxide production and peroxiredoxin 3 expression in the thyroid after excessive iodide exposure. MT-I/II may potentially protect the thyroid against high concentrations of iodide induced oxidative stress.

## Figures and Tables

**Table 1:** The median urinary iodine concentrations (*µ*g/L) of WT mice and MT-I/II KO mice.

Group	WT	MT-I/II KO
NI	320.29 ± 112.16	424.33 ± 131.95
10 HI	3592.92 ± 519.98^*^	3949.24 ± 647.93^*^
100 HI	30479.17 ± 4128.94^*^	40666.35 ± 3520.46^∗#^

^*^Compared to the NI group (*P* < 0.05); ^#^WT mice group compared to MT-I/II KO mice group (*P* < 0.05). *N* = 10 for each group.

**Table 2:** Changes of serum thyroid hormones level (nmol/L) in WT and MT-I/II KO mice following NI, 10 HI, or 100 HI intake for 14 days.

Group	T_3_	T_4_	FT_3_	FT_4_
WT				
NI	0.72 ± 0.13	26.08 ± 14.21	4.80 ± 0.42	28.56 ± 4.51
10 HI	0.53 ± 0.09	33.50 ± 4.45	4.26 ± 0.41	31.42 ± 0.95
100 HI	0.71 ± 0.13	34.70 ± 13.24	4.61 ± 0.25	33.20 ± 4.55
MT-I/II KO				
NI	0.58 ± 0.15	20.05 ± 4.68	4.04 ± 0.77	25.69 ± 0.97
10 HI	0.69 ± 0.13	22.53 ± 7.79	4.78 ± 0.05	27.22 ± 1.03
100 HI	0.80 ± 0.22	17.73 ± 6.76	5.01 ± 0.73	23.99 ± 1.92^*^

^*^In 100 HI group, compared to WT mice, there was a significant difference in serum thyroid hormones level (FT_4_) in MT-I/II KO mice (*P* < 0.05). *N* = 8 for each group.

**Table 3:** Documented literatures of iodide intake and changes of serum thyroxine (T_4_) and triiodothyronine (T_3_) concentrations.

Dosage of daily iodine intake	Time	Subject	Changes of serum T_4_, T_3_ concentrations	Reference
250 or 500 *μ*g	14 days	Normal volunteers	No change	[27]

1500 *μ*g	14 days	Normal volunteers	T_4_, T_3_ ↓ TSH ↑	[27]

50 or 250 mg	13 days	Normal subjects	T_4_, T_3_ ↓ TSH ↑ (within normal range)	[28]

27 mg	4 weeks	Normal volunteers	T_4_ within the normal range except for two subjects TSH ↑	[29]

Single doses of 10, 30, 50, and 100 mg and then daily doses of 10, 15, 30, 50, or 100 mg	12 days	Euthyroid volunteers	T_3_, T_4_ ↓ TSH ↑	[30]

190 mg	10 days	Euthyroid volunteers	T_3_, T_4_ ↓ TSH ↑	[31]

114 mg	3–7 weeks	Normal controls	T_3_, T_4_ ↓ TSH ↑	[32]

114 mg	3–7 weeks	Thyrotoxic patients	T_4_, T_3_ ↑ in some cases; other patients remained euthyroid even after 6 weeks	[32]

114 mg	2 weeks	Hypothyroid patients on thyroxine replacement	No consistent change (T_4_ ↑ at 1 and 14 days TSH ↓ the first day)	[32]

## Abbreviations

MT-I/II KO: Metallothionein I/II knockout
MTs: Metallothioneins
WT: Wild-type
ROS: Reactive oxygen species
Prx: Peroxiredoxin
KI: Potassium iodide
NI: Normal iodide intake
HI: High iodide intake
LDH: Lactate dehydrogenase
H_2_O_2_: Hydrogen peroxide
SOD: Superoxide dismutase
MTT: 3-(4,5-Dimethylthiazol-2-yl)-2,5-diphenyltetrazolium bromide
K-S: Kolmogorov-Smirnov
LSD: Least significant difference
T_4_: Thyroxine
T_3_: Tri-iodothyronine
FT_4_: Free thyroxine
FT_3_: Free tri-iodothyronine.
